# Burden of cancer in Malawi; common types, incidence and trends: National population-based cancer registry

**DOI:** 10.1186/1756-0500-5-149

**Published:** 2012-03-16

**Authors:** Kelias Phiri Msyamboza, Charles Dzamalala, Catherine Mdokwe, Steve Kamiza, Marshal Lemerani, Titha Dzowela, Damson Kathyola

**Affiliations:** 1 World Health Organisation, Malawi Country Office, Lilongwe, Malawi; 2 University of Malawi, College of Medicine, Histopathology Department, Blantyre, Malawi; 3 Ministry of Health, Lilongwe, Malawi; 4 World Health Organisation, Malawi Country Office, ADL House, 2nd Floor, City Centre, P.O. Box 30390 Lilongwe 3, Malawi

**Keywords:** Cancer, Non-communicable diseases, Sub-Saharan Africa, Malawi

## Abstract

**Background:**

Cancer is a leading cause of morbidity and mortality worldwide with a majority of cases and deaths occurring in developing countries. While cancer of the lung, breast, colorectum, stomach and prostate are the most common types of cancer globally, in east and southern Africa these are less common and comprehensive data to inform policies are lacking.

**Methods:**

Nationwide cancer registry was conducted between September and October 2010 in Malawi. New cancer cases registered from 2007 to 2010 were identified from hospital and clinic registers of 81 out of 84 health facilities providing cancer diagnosis, treatment or palliative care services. Demographic and cancer data were extracted from registers and case notes using a standard form.

**Results:**

A total of 18,946 new cases of cancer were registered in Malawi from 2007-2010. Of these 55.9% were females, 7.2% were children aged less than 15 years, 76.5% were adults aged 15-59 years and 16.4% were elderly aged 60 years or more. Only 17.9% of the cases had histologically verified diagnosis, 33.2% were diagnosed clinically and 49.6% based on clinical and some investigations. Amongst females, cancer of the cervix was the commonest accounting for 45.4% of all cases followed by Kaposi sarcoma (21.1%), cancer of the oesophagus (8.2%), breast (4.6%) and non-Hodgkin lymphoma (4.1%). In males, Kaposi sarcoma was the most frequent (50.7%) then cancer of oesophagus (16.9%), non-Hodgkin lymphoma (7.8), prostate (4.0%) and urinary bladder (3.7%). Age-standardised incidence rate per 100,000 population for all types of cancer in males increased from 31 in 1999-2002 to 56 in 2007-2010. In females it increased from 29 to 69. Kaposi sarcoma and cancer of the oesophagus, cervical cancer and Kaposi sarcoma were the main causes for the increased incidence in males and females respectively. It was estimated that, annually at least 8,151 new cases of cancer (all types) occur in Malawi.

**Conclusions:**

This study provided data on common types and trends of cancer that could be used to focus prevention, treatment and control interventions in the context of limited resources. The problem of under-reporting and misdiagnosis of cancer cases has been highlighted.

## Background

Cancer is a leading cause of morbidity and mortality worldwide. In 2008, globally, there were 12.7 million new cancer cases and 7.6 million cancer deaths (around 13% of all deaths) with 56% of the new cases and 63% of the cancer deaths occurring in developing countries. It is projected that by 2030, the number of new cancer cases and deaths will increase by 69% and 72% to 21.4 million and 13.2 million respectively [1,2]. While cancer of the lung (12.7%), breast (10.9%), colorectum (9.7%), stomach (7.8%) and prostate (7.2%) are the most common types of cancer globally [1,2], in sub-Saharan Africa (SSA) these are less common. In eastern and southern Africa (ESA) where HIV prevalence is high, AIDS-defining cancers including Kaposi sarcoma, cervical cancer, and non-Hodgkin lymphoma are the most common. In West Africa, cancer of the liver is the most common [3-6]. Prevalences of risk factors for cancer in ESA are high; HIV 10% or more, human papillomavirus 34%, tobacco smoking and harmful use of alcohol particularly in men 25% and 19% respectively and overweight in women 28% or more [7-9]. Indoor smoke from household use of solid fuels and urban air pollution are other common cancer risk factors in this region [10]. Comprehensive data on the burden and trends of cancer to inform policies, strategies and interventions are lacking in most countries in eastern and southern African region. Between September and October 2010, a nationwide cancer registry was conducted to determine the magnitude, trends of cancer and its challenges in Malawi.

## Methods

### Survey design, sites and data collection

Malawi Cancer Registry was established in 1985 as a population-based cancer registry with the aim of maintaining high quality cancer surveillance system and promoting research on cancer through collaboration. Utilising the cancer registry system, a cross-sectional health facility-based nationwide survey was conducted. All district (secondary), central (tertiary) and main public and private hospitals providing cancer laboratory diagnosis, treatment, palliative care or referral services were eligible and mapped out as survey sites. A total of 84 (70 public, 6 private, 6 research and 2 non-governmental) facilities met the criteria. Of these, 81 (94.4%) consented to take part and data were collected. Three facilities (all international research institutions) refused to take part. The reason for their refusal was that they needed clearance from their headquarters which was not granted. The other three research institutions were local and participated in this study. Cancer cases and deaths were identified from registers in outpatient department (OPD), inpatient wards (all wards available at the facility), clinics (all clinics available), laboratory, pharmacy, theatre and mortuary. Demographic and cancer data on the identified cancer cases or deaths were extracted using a standard form from Malawi Cancer Registry. Data were extracted for period 1 January 2007 to 31 August 2010. Data collection was conducted by trained health workers (nurses and clinical officers) from September to October 2010.

### Data management

Data were entered, cleaned and analysed using World Health Organisation cancer registry software version 4 (*Can Reg4*). The software had ability to detect duplicates thereby reducing the risk of double counting of cases that might have visited several hospitals or same hospital at different times. It also had the ability to estimate the age-standardised incidence rates based on age-specific national population data. National population data disaggregated by 5-year age groups and by sex was obtained from the Malawi National Statistics Office (NSO). The NSO data were entered and used to produce age-standardised incidence rates in *Can Reg4*. The existing data in the Malawi Cancer Registry database were used to produce trends of cancer from 1987-2010.

### Ethics statement

Ethical approval was granted by the Malawi National Health Sciences Research and Ethics Committee. Written informed consent to extract data from hospital registers and records was obtained from directors of hospitals that participated in the study.

## Results

### Characteristics of new cases of cancer and methods of diagnosis

For the period 1 January 2007 to 31 August 2010, a total of 18,946 new cases of cancer were registered in 81 out of 84 main health facilities (public, non-governmental organisations, and private) in Malawi. Of these new cases, 55.9% were females, 7.2% were children aged less than 15 years, 76.5% were adults aged 15-59 years and 16.4% were aged 60 years or more. Geographically, 13.4% of the new cases were from the northern, 35.0% from central and 51.6% from southern region. By diagnosis, only 17.9% of the new cases were diagnosed based on histology, cytology or haematology laboratory findings, 33.2% were diagnosed clinically (based on history taking and physical examination) and 49.6% based on clinical and some investigations (radiology, screening tests such as prostate specific antigen test, cervical cancer screening using visual inspection with acetic acid). Only 137 (0.7%) out of 18,946 cases had information on the stage of cancer at the time of diagnosis. Table 1 below shows number of registered new cases, method of diagnosis and geographical distribution of registered cancer cases in Malawi from 1987-2010.

**Table 1:** Number of registered new cases, method of diagnosis and geographical distribution of cancer in Malawi: 1987-2010

	1987-1990		1991-1994		1995-1998		1999-2002		2003-2006		2007-2010	
	**n**	**%**	**n**	**%**	**n**	**%**	**n**	**%**	**n**	**%**	**n**	**%**

**New registered cancer cases:**												

Males	2,335	46.4	2,276	49.5	2,576	51.3	3,983	50.5	6,850	49.8	8,360	44.1

Females	2,699	53.6	2,326	50.5	2,447	48.7	3,901	49.5	6,914	50.2	10,586	55.9

Total	5,034	100.0	4,602	100.0	5,023	100.0	7,884	100.0	13,764	100.0	18,946	100.0

**Method of cancer diagnosis:**												

Clinical only	0	0	405	8.8	924	18.4	2562	32.5	4515	32.8	6101	32.2

Clinical and some investigations	1	0.01	341	7.4	1000	19.9	2278	28.9	5602	40.7	9397	49.6

Histology/haematology/cytology	5033	99.9	3856	83.8	3099	61.7	3043	38.6	3647	26.5	3448	18.2

Total	5034	100.0	4602	100.0	5023	100.0	7884	100.0	13764	100.0	18946	100.0

**Cancer cases by region:**												

Northern	35	6.8	433	9.4	312	6.2	603	7.7	880	6.4	2538	13.4

Central	137	26.6	1,261	27.4	1,006	20.0	1,347	17.2	3,478	25.3	6,630	35.0

Southern	343	66.6	2,909	63.2	3,711	73.8	5,883	75.1	9,389	68.3	9,775	51.6

Total	515	100.0	4,603	100.0	5,029	100.0	7,833	100.0	13,747	100.0	18,944	100.0

n = number of registered new cancer cases,% = percentage

### Common types of cancer in Malawi

Of the 10,541 new cancer cases among females registered between 2007 and 2010, cancer of the cervix was the commonest accounting for 45.4% of all cases followed by Kaposi sarcoma (21.1%), cancer of the oesophagus (8.2%), breast (4.6%) and non-Hodgkin lymphoma (4.1%). In males, of the 8,314 new cases registered in the same period, Kaposi sarcoma was the commonest (50.7%) followed by cancer of oesophagus (16.9%), non-Hodgkin lymphoma (7.8%), prostate (4.0%) and urinary bladder (3.7%). In both sexes (sample size 18,855), the top five common cancers were; Kaposi sarcoma (34.1%), cancer of the cervix (25.4%), oesophagus (12.0%), non-Hodgkin lymphoma (5.7%) and urinary bladder (2.9%). Lung cancer was one of the least common accounting for only 0.2% of all cancers in both sexes, 0.2% and 0.1% in males and females respectively. In children under 15 years of age, of the 1,280 registered new cases, non-Hodgkin lymphoma (mainly Burkitt's) was the commonest accounting for 56.4% followed by Kaposi sarcoma (15.0%), cancer of the eye (7.3%), kidney (4.5%) and bone (2.2%). Of the 13,660 registered new cancer cases aged 15-59 years (both sexes), 41.3% had Kaposi sarcoma, 28.3% had cervical cancer, 9.4% had cancer of the oesophagus, 2.6% breast and 2.6% had eye cancer. In the elderly (age 60 years or more), common cancers were; oesophagus (28.7%), cervix (24.0%), Kaposi sarcoma (10.0%) and prostate (7.7%). Table 2 summarises the common types of cancer by gender and age.

**Table 2:** Common types of registered new cancer cases by gender and age in Malawi: 2007-2010

Cancer type	Males		Females		Both sexes		Children under 15 years old		Adults aged 15-59 years		Elderly aged 60 years or more	
	**%**	**(n)**	**%**	**(n)**	**%**	**(n)**	**%**	**(n)**	**%**	**(n)**	**%**	**(n)**

Lung	0.2	(19)	0.1	(14)	0.2	(33)	-	-	0.1	(19)	0.3	(10)

Breast	0.3	(26)	4.6	(480)	2.7	(506)	-	-	2.6	(357)	3.7	(109)

Kidney	0.4	(34)	0.4	(41)	0.4	(75)	4.5	(58)	0.1	(13)	0.2	(6)

Penis	1.4	(117)	-	-	0.6	(117)	-	-	0.6	(80)	0.9	(26)

Liver	1.5	(121)	0.7	(70)	1.0	(191)	1.5	(19)	0.9	(124)	1.2	(36)

Ovary	-	-	0.9	(99)	0.5	(99)	-	-	0.5	(72)	0.5	(15)

Bone	1.6	(129)	1.2	(123)	1.3	(252)	2.2	(28)	1.3	(174)	1.0	(29)

Stomach, intestine, rectum, anus	1.8	(147)	1.4	(152)	1.6	(299)	-	-	1.3	(179)	4.1	(121)

Eye	2.7	(225)	2.5	(266)	2.6	(491)	7.3	(94)	2.6	(352)	1.0	(30)

Urinary bladder	3.7	(308)	2.2	(232)	2.9	(540)	0.5	(7)	2.2	(300)	5.1	(149)

Prostate	4.0	(332)	-	-	1.8	(332)	-	-	0.5	(70)	7.7	(226)

Non-Hodgkin lymphoma	7.8	(649)	4.1	(427)	5.7	(1,076)	56.4	(722)	2.1	(289)	1.3	(37)

Oesophagus	16.9	(1,406)	8.2	(862)	12.0	(2,268)	-	-	9.4	(1,286)	28.7	(838)

Cervix	-	-	45.4	(4,787)	25.4	(4,787)	-	-	28.3	(3,869)	24.0	(701)

Kaposi sarcoma	50.7	(4,213)	21.1	(2,225)	34.1	(6,438)	15.0	(192)	41.3	(5,642)	10.0	(291)

Others	7.1	(588)	7.2	(763)	7.2	(1,351)	12.5	(160)	6.1	(834)	10.2	(297)

**Total**	**100.0**	**(8,314)**	**100.0**	**(10,541)**	**100.0**	**(18,855)**	**100.0**	**(1,280)**	**100.0**	**13,660**	**100.0**	**(2,921)**

n = number of cancer cases in the group,% = percentage

### Age-standardised incidence

**Figure 1:**
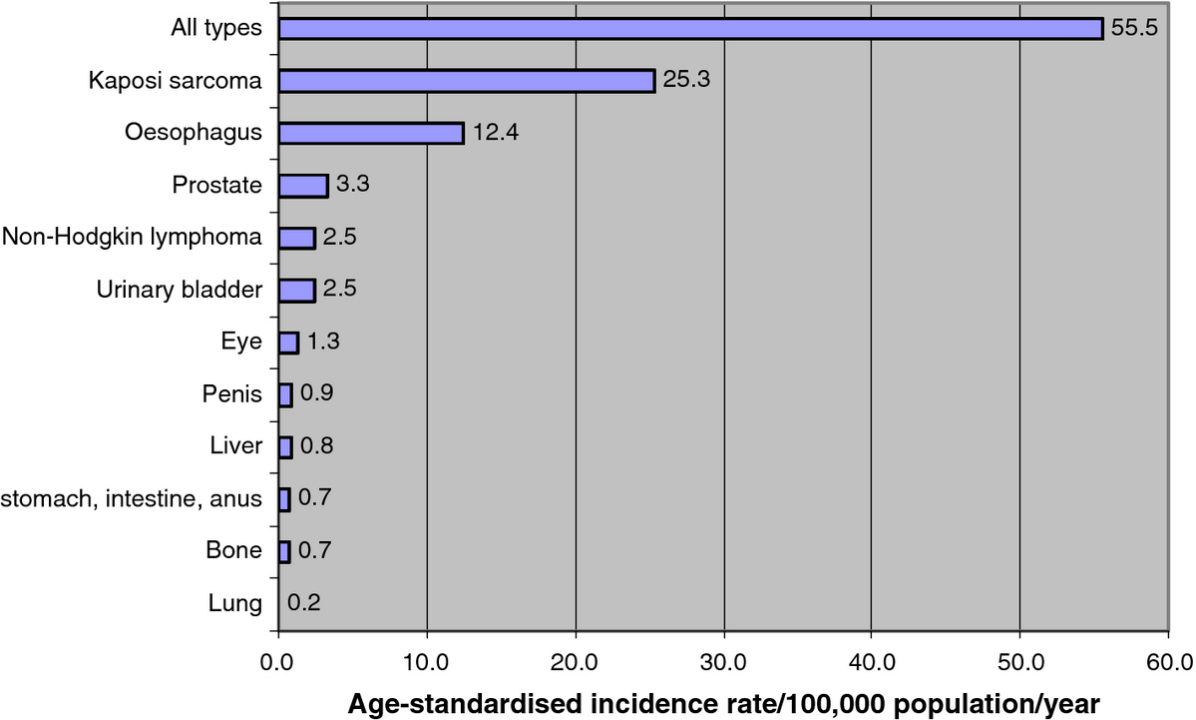
**Cancer age-standardised incidence rate in males in Malawi: 2007-2010**.

**Figure 2:**
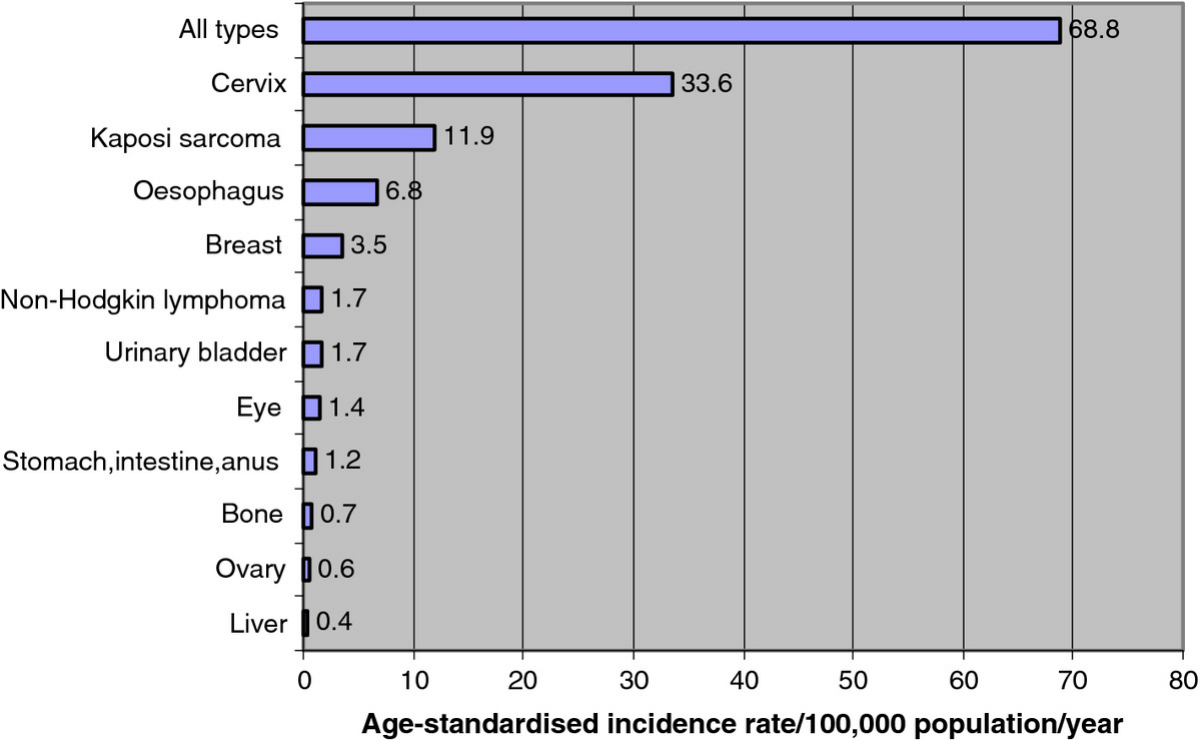
**Cancer age-standardised incidence rate in females in Malawi: 2007-2010**.

In males, age-standardised incidence rate (ASR) per 100,000 population per year was estimated at 55.5 while in females was 68.8 for all types of cancer. Kaposi sarcoma, cancer of the oesophagus, prostate, non-Hodgkin and urinary bladder were the top five cancers in males with ASR of 25.4, 12.4, 3.3, 2.5 and 2.5 respectively. In females, cancer of the cervix, Kaposi sarcoma, cancer of the oesophagus, breast and non-Hodgkin lymphoma were the top five with ASR of 33.6, 11.9, 6.8, 3.5 and 1.7 respectively. Based on these estimated ASR and age, sex disaggregated national population data from national statistical office, it was estimated that annually, there were at least 8,151 new cases of cancer (all types); 2,408 Kaposi sarcoma, 1,236 cervical cancer, 549 oesophageal cancer, 273 non-Hodgkin lymphoma, and 273 urinary bladder cancer cases in Malawi (Table 3, Figure 1 and Figure 2).

**Table 3:** Age-standardised incidence rate and estimated annual number of registered new cancer cases by gender in Malawi

	Age standardised cancer incidence rate per 100,000 population per year		Estimated annual number of new cancer cases		Estimated total annual number of new cancer cases
	**Males**	**Females**	**Males**	**Females**	

Lung	0.2	0.1	4	2	6

Penis	0.9	-	31	-	31

Liver	0.8	0.4	51	27	78

Ovary	-	0.6	-	40	40

Bone	0.7	0.7	45	47	92

Stomach, intestine, rectum, anus	0.7	1.2	19	36	55

Eye	1.3	1.4	83	94	177

Urinary bladder	2.5	1.7	159	114	273

Prostate	3.3	-	73	-	73

Non-Hodgkin lymphoma	2.5	1.7	159	114	273

Breast	-	3.5	-	106	106

Oesophagus	12.4	6.8	343	206	549

Kaposi sarcoma	25.4	11.9	1,609	799	2,408

Cervix	-	33.6	-	1,236	1,236

**All types**	**55.5**	**68.8**	**3,529**	**4,622**	**8,151**

### Trends of age-standardised incidence

**Figure 3:**
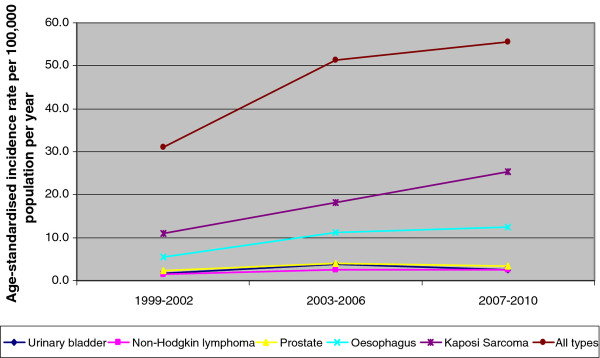
**Trends in cancer incidence in males in Malawi: 1999-2010**.

**Figure 4:**
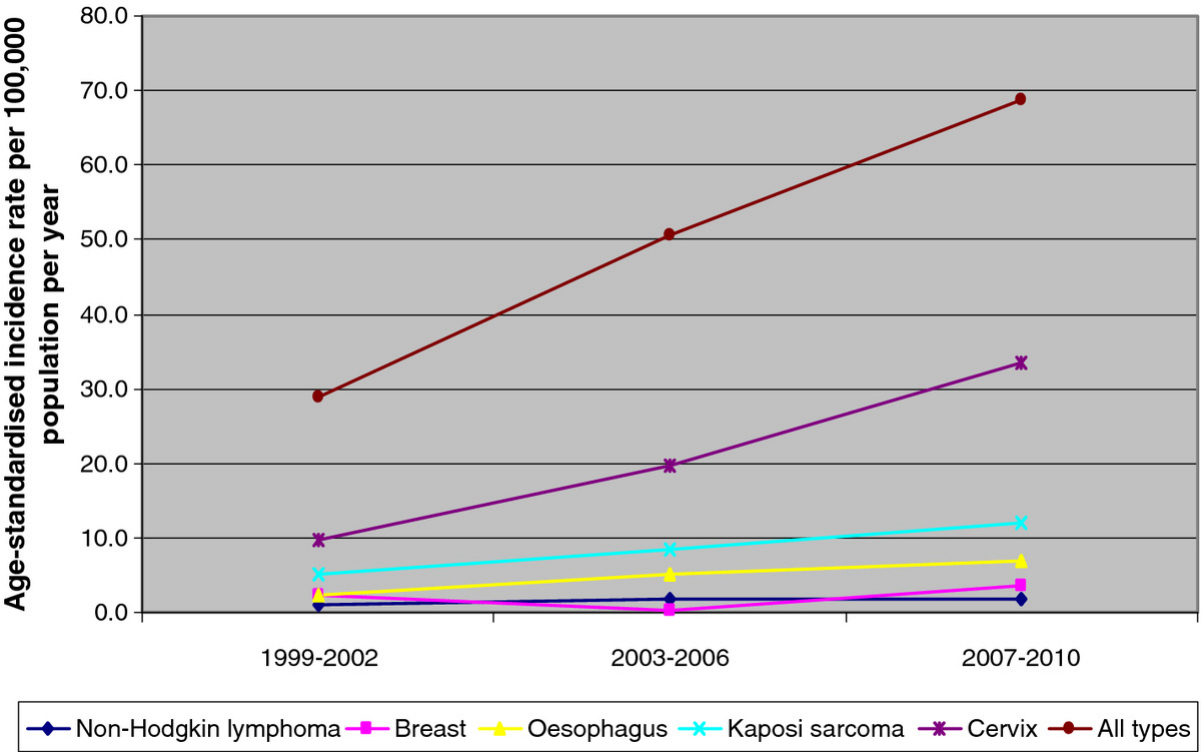
**Trends in cancer incidence in females in Malawi: 1999-2010**.

Age-standardised incidence of cancer per 100,000 population per year (all types) in males increased from 31.0 in 1999-2002 to 51.4 in 2003-2006 to 55.5 in 2007-2010. Kaposi sarcoma and cancer of the oesophagus had the highest increase in ASR which increased from 10.9 to 18.2 to 25.3; 5.5 to 11.1 to 12.4 respectively. In females, ASR increased from 28.8 to 50.5 to 68.8 for all types of cancer with the major increase in cervical cancer and Kaposi sarcoma of 9.8 to 19.6 to 33.6; 5.1 to 8.5 to 11.9 respectively (Figure 3 and Figure 4).

## Discussion

This national cancer registry study demonstrated that cancer is an important public health problem and incidence was increasing in Malawi. Kaposi sarcoma, cancer of the cervix and cancer of the oesophagus were the major cause of the increasing trend. With high HIV prevalence of 10.6% [11], the increase is Kaposi sarcoma and cervical cancer was in agreement with the findings from other studies [12,13]. The active case finding was more extensive in this survey providing more complete national data than previously reported.

The introduction and scaling up of free HIV antiretroviral therapy (ART) programme and insecticide-treated bednets (ITN) in 2003 did not seem to have an impact on the trends of Kaposi sarcoma, cervical cancer and Burkitt's lymphoma respectively as suggested by studies from other countries [13,14]. This could suggest that higher ART and ITN coverages are required to show impact on HIV and malaria related cancers than the current (2010) 65% coverage of estimated population in need of ART and 40% insecticide-treated bed net use in children under the age of five years [11,15]. For cancer of the oesophagus, it was not known why the trend was increasing. High HIV prevalence was less likely because the association between cancer of the oesophagus and HIV/AIDS is not well established although both may be related to fumonisin, a mycotoxin found in maize [16]. Increase in smoking, consumption of alcohol and maize contaminated with fumonisin could be some of the factors contributing to the increase in incidence of oesophageal cancer [7,16,17]. Lung cancer, the world's most frequent cancer in males, is generally reported to be less common in sub-Saharan Africa [3,18,19] although tobacco smoking is common with prevalence of at least 25% in men [7]. The reasons for relatively low prevalence of lung cancer in the presence of high prevalence of tobacco smoking in sub-Saharan Africa are unknown. Under-reporting, misdiagnosis, competing high levels of HIV/AIDS related cancers could be the possible explanations [18,20,21].

Our findings on the significance of gender and age differences for Kaposi sarcoma, cancer of oesophagus and non-Hodgkin lymphoma being more common in males than females, cancer of the oesophagus in people aged 60 years or more, and Kaposi sarcoma and non-Hodgkin lymphoma, in particular Burkitt's, being the most common cancers in children under 15 years of age were consistent with other studies [22-25]. Two thirds (67.1%) of non-Hodgkin lymphoma cases were children aged less than 15 years old. As countries in sub-Saharan Africa implement universal coverage of ART and ITNs, there would be need to monitor the trends of HIV and malaria related cancers as one way of assessing other public health benefits of these interventions.

The Malawi Ministry of Health, through Sexual and Reproductive Health Unit, implemented a screen-and-treat programme for cervical cancer using visual inspection with acetic acid (VIA) approach. The programme started in 2004 and targeted women aged 30-50 years old. As of June 2011, there were a total of 81 health facilities providing cervical cancer services (50 VIA only, 29 VIA and cryotherapy and 2 VIA, cryotherapy, loop electrosurgical excision procedure (LEEP) and major surgery). Cumulatively, a total of 59,217 women were screened, 5,744 (9.7%) were VIA positive and 1,777 (3.0%) had suspected cervical cancer [26]. This study demonstrated that 12.0% and 32.1% of cervical cancer cases were aged 20-29 years and 50 years or more respectively. This would suggest that the VIA Programme in Malawi could be missing at least 44% of women with cervical cancer. Data on population-based, age-specific prevalence of Human papillomavirus (HPV), the virus that causes cervical cancer is not available in Malawi. However, WHO estimates that the overall HPV prevalence is about 34% [27]. Studies from other countries reported HPV prevalence in women having two peaks, one at 15-29 year age group and the other at 60-69 year age group [28,29]. This, together with high HIV prevalence could be some of the reasons for cervical cancer occurrence in young and older age groups. Cervical cancer screening is the only established public health cancer screening programme in Malawi. Breast (mammography) and prostate (prostate-specific antigen test and digital rectal examination) cancer screening were available in only one private hospital. This private hospital participated in the study. Lung cancer was not routinely screened. Introduction of other cancer screening services in addition to cervical cancer in public health facilities could enhance early detection and treatment.

This study also revealed that only 18% of the cancer cases had laboratory verified diagnosis. Inadequate laboratory capacity (only one public and one private histology laboratory, all in Blantyre), long waiting time to get histology results, confidence of health workers in diagnosing some cancers clinically, in particular Kaposi sarcoma (the commonest cancer), late presentation of patients to health facility and lack of treatment, in particular, chemotherapy and radiotherapy could be some of the factors leading to low laboratory verified diagnosis. The low level of laboratory verified cases would suggest that the reported number of cancer cases was likely to be under-estimated because of under diagnosis and missing of cancer cases [21]. Current WHO estimates suggest that every year there are at least 2,316 new cases of cervical cancer cases in Malawi [27] while estimate from this study was 1,236. This illustrated that the reported number of registered new cervical cancer cases was only 53% of the actual problem at the community level. Some of the possible reasons for under-detection of cervical cancer cases could be low coverage of the screening programme (3.7% of the target group) and low specificity of VIA although it is the cost-effective screening method recommended for resource-limited settings and is comparable to Pap smear [27,30-32]. Despite the under-reporting, population-based cancer registry is the cost-effective method of collecting comprehensive data on cancer that resource-poor countries in sub-Saharan Africa are being encouraged to establish [33].

About half (52%) of registered new cancer cases (all types of cancer) were from the southern region with central and northern region accounting for 35% and 13% respectively. Population distribution was less likely to be the main reason as distribution was similar in the south and centre at 45% and 42% respectively [34]. The extent of active case finding in this survey was similar in all the regions whereby as many health facilities as possible were visited. Availability of diagnostic services hence less specimen transport cost and waiting time for results, cancer registry office and high HIV prevalence (south 18%, centre 10%, and north 8%) in the southern region were likely to be some of the contributing factors [11].

Data on lifestyles (tobacco smoking and alcohol consumption) and HIV status were not available. Some of the registers, particularly in public facilities were missing. These were the limitations of this study. These limitations apply to all studies that utilise available health facility data in general. Another limitation of this study was the refusal by three international research institutions to participate in this study. The reason for their refusal was that they had to seek clearance from their headquarters which was not granted. However, all the three international research institutions were housed within the public hospital and were recruiting cases from outpatient department or wards. In addition, all the institutions sent specimens to National Histopathology Laboratory which participated in this study. It was therefore less likely that non-participation of these three international research institutions had significant effect on the results as most of cases were likely to be captured either from public hospital registers where cases were first registered or from histology laboratory where specimens were sent or both. The use of clinical diagnosis only, cancer screening tests such as VIA and prostate specific antigen (PSA) have their own limitations but these limitations were beyond this study. This study only collected and analysed data that were already there. Nevertheless, the comprehensive analysis of the burden of cancer and its challenges demonstrated by this study provided local evidence that could be used to inform policies, strategies and interventions for prevention and control of cancer in Malawi, eastern and southern African region.

## Conclusion

Cancer is a public health problem and the trend is increasing in Malawi, a poor country in eastern and southern Africa. Scaling up and/or improving the quality of cervical and breast cancer screening programmes, laboratory verified diagnosis, chemotherapy and radiotherapy treatment and introduction of human papillomavirus vaccine for cervical cancer control could be some of cancer prevention, treatment and control interventions that Malawi and other countries in eastern and southern Africa may consider to strengthen or adopt.

## Competing interests

The authors declare that they have no competing interests.

## Authors' contributions

Conceived and designed the study: KPM, CD, CM. Performed the study: KPM, CD, CM, SK, TD, ML, DK. Analyzed the data: KPM, CD, CM. Contributed reagents/materials/analysis tools: KPM, CD. Wrote the paper: KPM, CD, CM, SK, TD, ML, DK. All authors read and approved the final manuscript.
